# Early re-emerging tremor after MRgFUS thalamotomy: case–control analysis of procedural and imaging features

**DOI:** 10.3389/fneur.2024.1356613

**Published:** 2024-06-06

**Authors:** Federico Bruno, Pierfrancesco Badini, Antonio Innocenzi, Gennaro Saporito, Alessia Catalucci, Patrizia Sucapane, Antonio Barile, Ernesto Di Cesare, Carmine Marini, Francesca Pistoia, Alessandra Splendiani

**Affiliations:** ^1^Department of Biotechnological and Applied Clinical Sciences, University of L'Aquila, L'Aquila, Italy; ^2^Neuroradiology and Interventional Radiology, San Salvatore Hospital, L’Aquila, Italy; ^3^Neurology, San Salvatore Hospital, L’Aquila, Italy

**Keywords:** essential tremor, Parkinson’s disease, MRgFUS, MRI, tremor recurrence

## Abstract

**Purpose:**

This study aimed to identify possible prognostic factors determining early tremor relapse after Magnetic Resonance guided Focused Ultrasound Surgery (MRgFUS) thalamotomy in patients with essential tremor (ET) and Parkinson’s disease (PD).

**Methods:**

Nine patients (six ET and three PD) who underwent Vim MRgFUS thalamotomy in a single institution and developed early re-emergent tremor were analyzed. A control group of patients matched pairwise for sex, pathology, age, disease duration, and skull density ratio (SDR) was selected to compare the technical-procedural data and MR imaging evidence. MR imaging findings compared between groups included lesion shape and volume in multiparametric sequences, as well as Fractiona Anisotropy (FA) and Apparent Diffusion Coefficient (ADC) values derived from Diffusion Tensor Imaging Diffusion Weighted Imaging (DTI) and Diffusion Weighted Imaging (DWI) sequences.

**Results:**

We did not find statistically significant differences in gender and age between the two groups. Technical and procedural parameters were also similar in both treatment groups. In MRI analysis, we found lesions of similar size but with greater caudal extension in the control group with stable outcomes compared to patients with tremor relapse.

**Conclusion:**

In our analysis of early recurrences after thalamotomy with focused ultrasound, there were neither technical and procedural differences nor prognostic factors related to lesion size or ablation temperatures. Greater caudal extension of the lesion in patients without recurrence might suggest the importance of spatial consolidation during treatment.

## Introduction

Vim thalamotomy using focused ultrasound is a well-established method for the treatment of Parkinson’s and essential tremor (ET) (1–3). Numerous studies have confirmed the indications, clinical findings, and complications. The results of efficacy in reducing tremors with follow-up up to 5 years are now available, with a known occurrence of recurrence in approximately 10% of cases (4–6). Many cases have also been retreated with the method or subsequently subjected to Deep Brain Stimulation (DBS) to consolidate the result (7–9). Although numerous factors that contribute to successful long-term treatment have been proposed and identified, including in previous studies by the authors, what is a common experience in many centers is the possible occurrence of tremor reoccurrence very early, within 1 month of treatment. In these cases, the determining factors often remain unclear (8, 10, 11).

Therefore, our study aimed to evaluate the influence of procedural and imaging parameters on the early recurrence of tremor in patients submitted to MRgFUS Vim thalamotomy, compared to those with a sustained optimal outcome.

## Materials and methods

We retrospectively evaluated all patients submitted to MRgFUS Vim thalamotomy at our institution between March 2018 and January 2023. From clinical reports, we retrieved patients with early tremor relapse (It is defined as an increase in the Fahn–Tolosa–Martin (FTM) part A score of ≥3 points after the post-procedural clinical assessment at 24 h.) that occurred within 1 month after treatment. According to our protocol, all patients are subjected to clinical and instrumental follow-up 1 day, 1 month, and 6 months after treatment.

All procedures were performed as explained in detail in other publications (1). In particular, Vim targeting was performed with indirect coordinates as follows:

- Halfway between one-third and one-fourth of the Anterior Commissure - Posterior Commissure (AC-PC) distance from the PC.
- Halfway between 14 mm from the AC-PC line and 11 mm from the lateral wall of the third ventricle.
- 2 mm above the AC-PC line.

In all patients, we recorded clinical-demographic features, procedural data, and MR findings. Patients with missing or incomplete clinical data, procedural reports, and MRI follow-up were excluded.

Clinical and demographic characteristics included as follows: underlying pathology, age, gender, disease duration, and skull density ratio (SDR).

Procedural data were retrieved from treatment reports and were included as follows:

- Ablative sonications, i.e., the number of sonications performed during the treatment reaching a mean target temperature of ≥54°C.
- Mean temperature (°C), i.e., the highest value of mean temperature reached during sonications.
- Maximum temperature (°C), i.e., the highest value of maximum temperature reached during sonications.

Imaging evaluation included the measurement of the lesion size and shape at the thalamus level, expressed in millimeters, measured as the maximum diameter on Fluid Attenuation Inversion Recovery (FLAIR), T1, T2, Susceptibility Weighted Imaging (SWI), and DWI-weighted sequences in the axial plane. For the evaluation of the shape, on the coronal sequences, the lesion cranial and caudal extension were measured in millimeters with respect to the AC-PC plane. In the same plane as the spatial measurements, an ROI was placed on the thalamotomy lesion for the quantitative measurement of FA and ADC values, respectively, in DWI- and DTI-weighted sequences. All MRI examinations were performed using a 3-Tesla MR-scanner (MR750w, GE Healthcare) with a 32-channel head coil. Acquisition parameters were as follows: slice 3.0–0.3, TR 7854, freq. FOV 26, and phase FOV 0.8. The same MRI protocol was applied for the follow-up examinations at 24 h, 1 month, and 6 months after treatment. Thalamotomy lesions were manually measured on a PACS workstation (Vue Motion, Carestream Health) by two neuroradiologists (AC, FB, with 16 and 4 years of experience in neuroimaging, respectively) using a digital ruler tool. The slice at the thalamus level that showed the greatest extent of the lesion and edema was chosen. Both readers were blinded to clinical and procedural information.

All procedural and imaging data were compared with a selected control group of patients without tremor relapse at the same follow-up interval, matched pairwise for age, sex, pathology, years of disease, pre-treatment FTM score, and SDR values (Table 1).

**Table 1:** Clinical data of the study population and control group.

	Study group	Control group
Sex (M/F)	8/1	8/1
Pathology (ET/PD)	6/2	6/2
Disease Duration	10.67 ± 5.92 (5–20)	9.89 ± 6.68 (6–18)
Age	68.44 ± 10.38 (47–74)	67.52 ± 11.23 (45–76)
SDR	0.45 ± 0.09 (0.35–0.58)	0.47 ± 0.11 (0.38–0.56)

### Statistical analysis

Data analyses were performed by using XLSTAT 2017: Data Analysis and Statistical Solution for Microsoft Excel (Addinsoft, Paris, France, 2017). Qualitative variables were summarized as frequency and proportions. Values of continuous variables were tested for normal distribution with Shapiro–Wilk’s test and reported as mean and standard deviation (SD) or median and interquartile range (IQR), according to their distribution. Differences in quantitative values between groups were compared using the *t*-test or Wilcoxon test.

## Results

Out of a total of 175 patients treated during the study period, 9 patients (8 men, mean age 68.44 ± 10.38 years) showed evidence of early tremor relapse. All patients had been treated in the right hand with left thalamotomy. No adverse effects or complications were recorded in all patients at the time of follow-up.

The clinical characteristics of the study group are summarized in Table 1.

As illustrated in Table 2, the analysis of the trend of the assessment of tremor intensity through the FTM scale demonstrated a reduction in tremor part A of approximately 85% at 24 h, reduced at 1-month follow-up to 78%. In part B of the tremor, there was a reduction of approximately 30% at 24 h and then reduced to 7% at 1 month.

**Table 2:** Tremor intensity trend with FTM scale (treated side).

	FTM
Pre tot	44.18 ± 11.93 (27–68)
Pre part A	13.36 ± 4.27 (7–22)
Pre part B	16.01 ± 6.54 (6–24)
24 h tot	25.73 ± 8.96 (12–43)
24 h part A	7.55 ± 3.72 (2–13)
24 h part B	11.64 ± 4.84 (4–19)
1mo tot	31.36 ± 9.43 (19–51)
1mo part A	10.45 ± 3.27 (5–16)
1mo part B	15.18 ± 5.17 (9–24)

**Figure 1:**
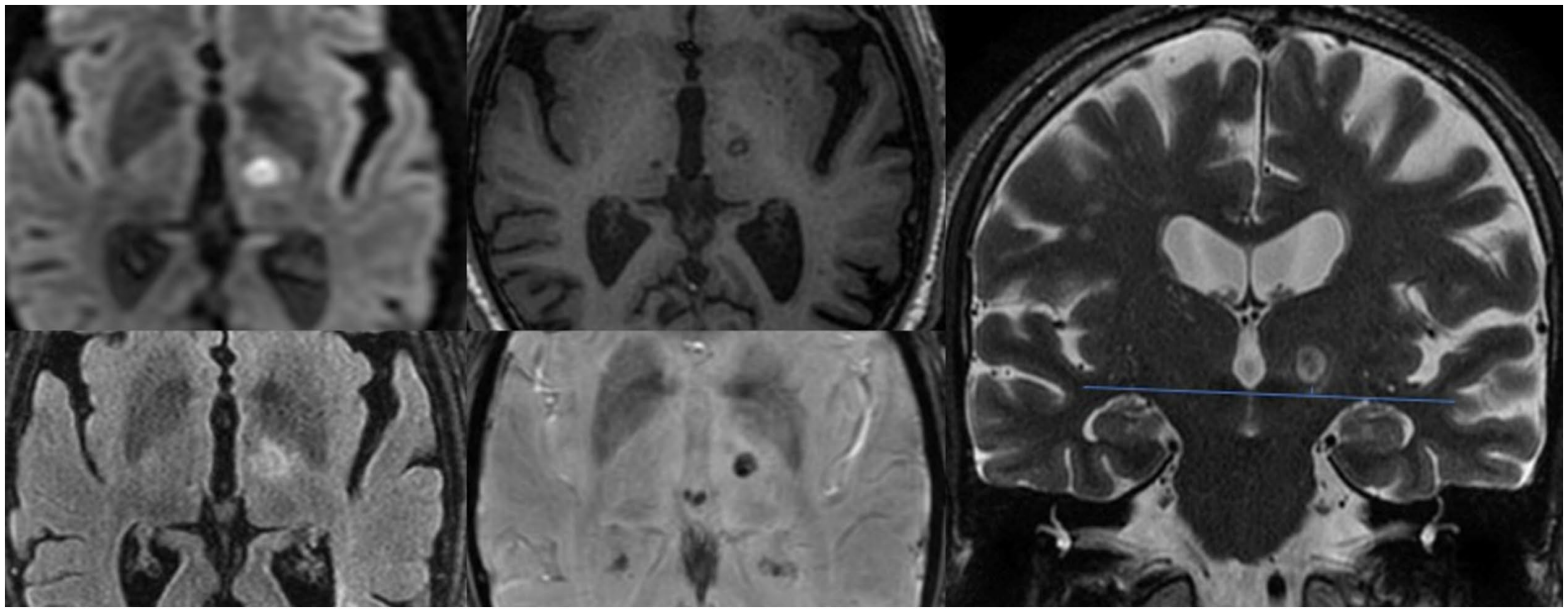
Evaluation of thalamotomy lesion axial plane diameter on DWI, FLAIR, T1 and SWI sequences, and caudal extension with respect to the AC-PC plane visualized on coronal T2 images.

In both groups, we found a progressive decrease in the thalamotomy lesion size (Table 3). In assessing the size of the lesion, we also considered the total brain volume of the patients, which showed no statistically significant differences between the two study groups (1397.22 ± 74.64 mL in the study group vs. 1403.25 ± 59.25 mL in the control group, *p* = 0.855). However, we did not find significant size differences between the study (relapse) group and the controls. In the analysis of the lesion shape, patients without recurrence showed a more elongated shape, with significantly more caudal extension below the AC-PC (*p* = 0.02) (Table 3 and Figure 1).

**Table 3:** Thalamotomy lesion size at 1 month.

	Study group	Control group	*p*-value
T1 1mo	5.11 ± 1.45 (3–7)	5.22 ± 1.39 (4–8)	0.253
FLAIR 1mo	6.22 ± 1.3 (4–8)	6.44 ± 1.33 (5–8)	0.365
T2 1mo	5.67 ± 1.22 (3–7)	6.22 ± 1.72 (4–9)	0.625
DWI 1mo	6.11 ± 1.05 (5–8)	6.67 ± 1.41 (5–9)	0.732
SWI 1mo	6.11 ± 1.62 (4–9)	6.56 ± 1.94 (2–8)	0.196
AC-PC 1mo	1.83 ± 0.87 (0.5–3)	0.44 ± 1.74 (−3–2)	**0.021**

Statistically significant results in bold.

Quantitative evaluation of ADC values demonstrated the presence of residual signal restriction with decreased ADC values in both groups. There was also a decrease in AF values in both cohorts, which was statistically lower in the study group than in the control group (Table 4).

Regarding the analysis of procedural data, we did not find statistically significant differences between the two groups (Table 5).

**Table 4:** ADC and FA values at 1 month.

	Study group	Control group	*p*-value
ADC 1mo	0.55 ± 0.16 (0.33–0.85)	0.56 ± 0.13 (0.36–0.71)	0.978
FA 1mo	0.13 ± 0.03 (0.08–0.17)	0.25 ± 0.11 (0.12–0.41)	**0.008**

Statistically significant results in bold.

**Table 5:** Procedural and sonication parameters in the study and control group.

	Study group	Control group	*p*-value
Ablative sonications (N)	2.38 ± 1.06 (1–4)	2.86 ± 1.57 (1–5)	0.979
Mean temperature (°C)	55.75 ± 1.67 (53–58)	54.86 ± 1.21 (1–41)	0.689
maximum temperature (°C)	61.38 ± 1.85 (58–64)	58.43 ± 0.98 (57–60)	0.715

## Discussion

The occurrence of extremely early recurrences is the common experience of many centers performing high-volume MRgFUS; however, still, limited information is discussed in the literature except in a few case reports (4, 7–9).

In fact, most of the recurrences described in trials and observational studies involve those arising within 6–12 months, which is known to occur in approximately 10–11% of cases. Some factors influencing this type of recurrence, which can also be partially considered a “loss of efficacy,” involve demographic factors, primarily the underlying pathology, where tremor recurrence is more frequent in patients affected by Parkinson’s disease (PD) or long-standing essential tremor (ET) before developing PD symptoms. In our cases with early recurrence, however, only two were affected by PD (1, 2, 5, 6, 12–14).

According to numerous authors, including our previous experience, it is crucial to consider the size of the lesion in order to achieve a durable and established outcome. In our cohort, there were no statistically significant differences in lesion size at 1 month. However, a noteworthy imaging finding was the caudal extent of the lesion. This finding is particularly intriguing. It is a common approach for many centers to set the initial coordinates of their target at 2 mm above the AC-PC plane in order to minimize the risk of adverse effects. Nevertheless, lesions that are positioned too high in relation to the AC-PC plane appear to be more closely linked to recurrence (14–17).

No dissimilarities in lesion size were detected between the two groups during the MRI follow-up after 1 month. This contrasts partially with the findings of Atkinson et al.’s study, which revealed that patients who achieved excellent post-treatment outcomes displayed larger lesions. Nevertheless, in both groups, the lesion size—measured as the maximum diameter—fell within the normal range when compared to the accepted standards for a sufficiently ablative lesion (18–20).

Some previous studies in the literature have evaluated changes in DWI and DTI metrics after MRgFUS. In particular, it is known that at the lesion level, there is evidence of necrosis with the restriction of diffusivity and reduction of ADC values. The changes in FA values measured at the level of the Vim could be indicative of the actual disruption of the fiber bundles involved in tremor and in particular, the dentato-rubro-thalamic tract (DRTT) bundle (21). In the paper by Hori et al., researchers found that TcMRgFUS thalamotomy resulted in a significant decrease in relative FA (rFA) values in the targeted Vim at 1 day and 1 year after treatment. These changes in rFA values also showed a significant correlation with clinical outcomes measured by the Clinical Rating Scale for Tremor scores at 1 year follow-up. This implies that FA may be a potential imaging biomarker for early prediction of clinical outcomes after TcMRgFUS thalamotomy for ET (10).

In partial disagreement with what was expected, in our study, we did not show a lower reduction in FA values compared with patients in the control group.

Some other previous observations suggest that the disruption of the DRTT is only partially a prognostic element of stable tremor reduction. According to Maamary et al., who reported two instances of early tremor recurrence in PD patients following MRgFUS thalamotomy, the disruption of the DRTT may only partially and temporarily halt tremor outflow, allowing other circuits, particularly the PTT, to persist in propagating tremors. A possible explanation for the recurrence of tremors following VIM thalamotomy is that although the interruption of major pathways, such as the DRTT, initially suppresses tremors, re-routing through unaffected parts of the tremor network, specifically the PTT, could potentially lead to tremor recurrence (8, 22, 23).

The above would not only be applicable to Parkinson’s tremors, in which recurrence is more frequent in the literature than in ETs but also in the latter, which were found to account for the majority of recurrences in the present study. Indeed, the experience of Gallay et al. in performing cerebellothalamic tractotomy, ablation with a target placed 3 mm below the ICP was found to have improved target coverage and procedural efficacy, with tremor relief of up to 90% at 1 year follow-up (23).

The hypothesis is bolstered by various factors, such as the case where repeat Vim MRgFUS thalamotomy did not offer additional advantages, but targeting the subthalamic area proved to be effective (8, 17). Furthermore, there have been inconsistent findings regarding lesion overlap and the visualization of the DRTT bundle after thalamotomy, both in patients with recurrence and in those with stable outcomes.

Accuracy in targeting, the operator’s experience with the method, and intraoperative monitoring are key factors in achieving an ablative and established lesion (14, 24–26).

In a recent commentary, Önder proposed a hypothesis regarding the recurrence of tremors after MRgFUS thalamotomy in PD patients. The hypothesis suggests that the histopathological effects of MRgFUS treatment may differ from other techniques, such as RF and gamma knife thalamotomy. Specifically, a unique post-mortem histopathological examination of a patient who underwent MRgFUS revealed demyelination, abundant lipid-laden macrophages, and relatively preserved neurons and axons in the lesion. Therefore, MRgFUS is hypothesized to preferentially cause demyelination rather than necrosis. It is suggested that the decline in the benefit of MRgFUS on tremors over time in PD patients may be related to possible amelioration of the demyelinating injury (27).

Although MRgFUS is a repeatable technique in cases of recurrence, it, therefore, remains to be clarified what is the best strategy used for targeting in these cases, whether to consider vim recentering by imaging and direct targeting or to choose another target, or to prefer a different method such as DBS (7, 27, 28).

The current research has certain limitations that warrant acknowledgment. First, the sample size of the study group is modest, given that the incidence of relapse after MRgFUS thalamotomy is relatively low. Additionally, the follow-up duration was restricted to only 1 month. Conducting future studies with a larger participant pool and an extended follow-up duration may be advantageous to validate our findings.

## Data availability statement

The raw data supporting the conclusions of this article will be made available by the authors, without undue reservation.

## Ethics statement

Written informed consent was obtained from the individual(s) for the publication of any potentially identifiable images or data included in this article.

## Author contributions

FB: Conceptualization, Data curation, Formal analysis, Funding acquisition, Investigation, Methodology, Project administration, Resources, Software, Supervision, Validation, Visualization, Writing – original draft, Writing – review & editing. PB: Conceptualization, Data curation, Formal analysis, Funding acquisition, Investigation, Methodology, Project administration, Resources, Software, Supervision, Validation, Visualization, Writing – original draft, Writing – review & editing. AI: Conceptualization, Data curation, Formal analysis, Funding acquisition, Investigation, Methodology, Project administration, Resources, Software, Supervision, Validation, Visualization, Writing – original draft, Writing – review & editing. GS: Conceptualization, Data curation, Formal analysis, Funding acquisition, Investigation, Methodology, Project administration, Resources, Software, Supervision, Validation, Visualization, Writing – original draft, Writing – review & editing. AC: Conceptualization, Data curation, Formal analysis, Funding acquisition, Investigation, Methodology, Project administration, Resources, Software, Supervision, Validation, Visualization, Writing – original draft, Writing – review & editing. PS: Conceptualization, Data curation, Formal analysis, Funding acquisition, Investigation, Methodology, Project administration, Resources, Software, Supervision, Validation, Visualization, Writing – original draft, Writing – review & editing. AB: Funding acquisition, Investigation, Methodology, Project administration, Resources, Software, Supervision, Validation, Visualization, Writing – original draft, Writing – review & editing, Conceptualization, Data curation, Formal analysis. EC: Conceptualization, Data curation, Formal analysis, Funding acquisition, Investigation, Methodology, Project administration, Resources, Software, Supervision, Validation, Visualization, Writing – original draft, Writing – review & editing. CM: Conceptualization, Data curation, Formal analysis, Funding acquisition, Investigation, Methodology, Project administration, Resources, Software, Supervision, Validation, Visualization, Writing – original draft, Writing – review & editing. FP: Conceptualization, Data curation, Formal analysis, Funding acquisition, Investigation, Methodology, Project administration, Resources, Software, Supervision, Validation, Visualization, Writing – original draft, Writing – review & editing. AS: Conceptualization, Data curation, Formal analysis, Funding acquisition, Investigation, Methodology, Project administration, Resources, Software, Supervision, Validation, Visualization, Writing – original draft, Writing – review & editing.
